# The Cockayne syndrome protein B is involved in the repair of 5-AZA-2′-deoxycytidine-induced DNA lesions

**DOI:** 10.18632/oncotarget.26189

**Published:** 2018-10-12

**Authors:** Estefanía Burgos-Morón, José Manuel Calderón-Montaño, Nuria Pastor, Andreas Höglund, Ángel Ruiz-Castizo, Inmaculada Domínguez, Miguel López-Lázaro, Nabil Hajji, Thomas Helleday, Santiago Mateos, Manuel Luis Orta

**Affiliations:** ^1^ Department of Cell Biology, Faculty of Biology, University of Seville, 41012 Seville, Spain; ^2^ Department of Pharmacology, Faculty of Pharmacy, University of Seville, 41012 Seville, Spain; ^3^ Science for Life Laboratory, Division of Translational Medicine and Chemical Biology, Department of Medical Biochemistry and Biophysics, Karolinska Institute, S-171 21 Stockholm, Sweden; ^4^ Department of Medicine, Division of Experimental Medicine, Centre for Pharmacology & Therapeutics, Toxicology Unit, Imperial College London, Hammersmith Campus, London, W12 0NN UK; ^5^ Present address: Sprint Bioscience AB, 141 57 Huddinge, Sweden

**Keywords:** CSB, 5-azadC, DNMT1, DNA damage, transcription

## Abstract

The Cockayne Syndrome Protein B (CSB) plays an essential role in Transcription-Coupled Nucleotide Excision Repair (TC-NER) by recruiting repair proteins once transcription is blocked with a DNA lesion. In fact, CSB-deficient cells are unable to recover from transcription-blocking DNA lesions. 5-Aza-2′-deoxycytidine (5-azadC) is a nucleoside analogue that covalently traps DNA methyltransferases (DNMTs) onto DNA. This anticancer drug has a double mechanism of action: it reverts aberrant hypermethylation in tumour-suppressor genes, and it induces DNA damage. We have recently reported that Homologous Recombination and XRCC1/PARP play an important role in the repair of 5-azadC-induced DNA damage. However, the mechanisms involved in the repair of the DNMT adducts induced by azadC remain poorly understood. In this paper, we show for the first time the importance of CSB in the repair of azadC-induced DNA lesions. We propose a model in which CSB initiates a signalling pathway to repair transcription blocks induced by incorporated 5-azadC. Indeed, CSB-deficient cells treated with 5-azadC show a delay in the repair of trapped DNMT1, increased levels of DNA damage and reduced survival.

## INTRODUCTION

After millions of years of evolution, cells have evolved complex mechanisms to repair DNA breaks and prevent mutations. Although it has been known for many years that transcriptional stress plays an important role in genomic instability [1–5], it was in the mid-eighties when an additional DNA repair mechanism associated with transcriptionally active genes [6–7] was discovered. This specialized DNA repair processes, called Transcription-Coupled Repair (TCR), couples RNA polymerase blocks with the efficient removal of DNA lesions in the transcribed strand. This pathway is considered as a branch of the nucleotide excision repair pathway (NER). In humans, mutations in NER lead to a variety of DNA repair disorders, including Cockayne syndrome (CS), in which there is a deficiency in TCR. Two complementation groups of CS, designed CSA and CSB have been identified. Cells with mutations in any of these proteins cannot resume transcription after the UV-induced blockage of RNA polymerase [8, 9].

CSB is a 168 kDa protein related to the SWI/SNF family of ATP-dependent chromatin remodelers; this protein has nucleosome remodeling activity and binds to core histone proteins *in vitro*. When transcription fork is blocked, CSB protein is recruited and strongly interacts with RNA pol II. This protein acts as a chromatin remodeling factor displacing nucleosomes and recruiting some protein complexes, including the CSA complex, core NER factors (XPA, TFIIH, XPG, XPF-ERCC1, and RPA) and histone acetyltransferase p300 (that also works as a chromatin remodeling factor) [8]. The CSA complex acts by ubiquitination and subsequent degradation of CSB, RNA pol II, CSA itself and histones [10]. This clearance of proteins is needed for DNA repair and subsequent resumption of transcription. Apart from its roles in transcription coupled nucleotide excision repair (TC-NER) and chromatin remodeling, CSB is thought to be involved in oxidative damage [11], crosslink repair [12], telomere maintenance [13], transcription associated DNA recombination [14], double strand break repair choice and checkpoint activation [15].

5-aza-2′-deoxycytidine (5-azadC), also called decitabine, is a cytidine analogue that is incorporated randomly in the genome during replication. This drug is effective in the treatment of Myelodysplastic Syndromes and Acute Myeloid Leukemia (AML), this latter especially in elderly patients [16, 17]. Its mechanism of action involves the covalent trapping of DNA methyltransferases (DNMTs) onto DNA, generating a whole hypomethylation state [18]. Therefore, this drug can reactivate the expression of Tumour Suppressor Genes whose promoters are highly hypermethylated [19]. Trapped DNMTs onto DNA generate DNA damage, which also contributes to the anticancer properties of this nucleoside [20–22].

The mechanisms involved in the repair of the DNMT adducts induced by azadC remain poorly understood. We recently reported that these bulky lesions can interfere with replication forks and induce double strand breaks (DSBs) that are repaired by Homologous Recombination (HR) involving Fanconi Anemia (FA) proteins (21). Also, we have proposed that XRCC1 and PARP could play a role in the repair of DNMT adducts [22]. In the present paper, we investigate the role of CSB in the repair of the lesions induced by 5-azadC. We show that CSB is important in the repair of the lesions induced by 5-azadC in a process that is independent of classic TC-NER. We found that a transcription coupled DNA damage response (TC-DDR) is activated shortly after 5-azadC incorporation in a CSB dependent manner. Furthermore, our results revealed that CSB-deficient cells displayed a delay in the repair of DNMT1 adducts, resulting in hypersensitivity to 5-azadC. Finally, we demonstrate that CSB and transcription act in the same pathway to repair 5-azadC-induced DNA lesions and promote survival.

## RESULTS

### 5-azadC induces transcription-dependent double strand breaks

**Figure 1:**
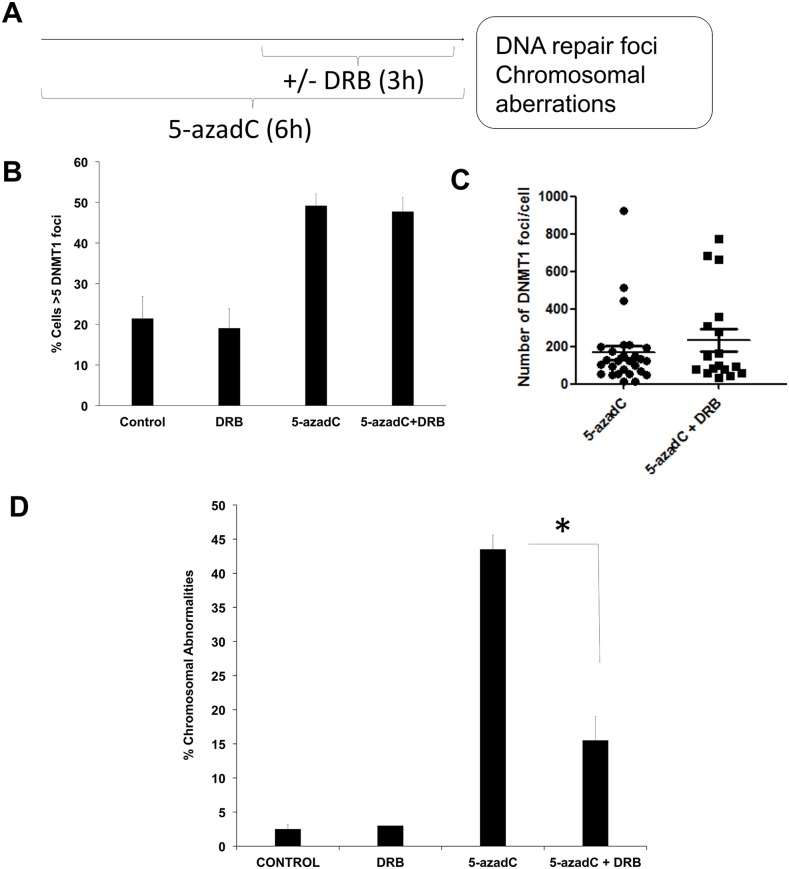
5-azadC induces transcription-coupled DSBs **(A)** Experimental schedule followed. AA8 cells were treated for 6h with 15 μM of 5-azaC. DRB (30μM) was present during the last 3h. **(B)** In order to evaluate DNMT1 foci, DNMT1-GFP transfected cells were treated as described above, fixed in paraformaldehyde and scored for DNMT1 foci. Cells containing >5 DNMT1 foci were scored as positive. **(C)** Alternatively the number of DNMT1 foci was scored using Image J software. **(D)** In order to analyze chromosomal abnormalities after 5-azadC treatment (50μM), cells were treated with colcemid and metaphase spreads were made as described in the material and methods section. The means and SEM of three independent experiments are shown. Data were statistically analyzed using Student's *t*-test. Data were considered statistically different when P <0.05 (^*^) or P <0.01 (^**^).

We have recently reported that 5-azadC induces DNA damage that depends on active replication, which suggests that trapped DNMT collapses with oncoming replication forks into DSBs (21). Here we wanted to investigate the possible role of transcription elongation in the generation of DNA breaks after 5-azadC treatment. In order to distinguish between replication and transcription-associated DNA DSBs, we exposed the cells to 5-azadC for 6 h. This short exposure time impossibilities that a second round of DNA replication takes place over a 5-azadC substituted DNA (Figure 1A).

DNA damage was monitored by the scoring of chromosome aberrations as indicators of DSBs [23]. Cells were co-treated with the transcription inhibitor DRB during the last 3 h to see if transcription elongation is required for the formation of chromosomal abnormalities. Under these conditions, when DNMT1-GFP transfected AA8 cells were treated with 5-azadC, they presented increased levels of DNMT1 foci as compared to control cells (Figure 1B–1C). Of interest, we could not find any significant difference in the levels of trapped DNMT1 between 5-azaC treated and co-treated cells. This data indicates that similar levels of the initial lesions are present in both cases.

It is worth mentioning that a 6 h treatment with 5-azadC induced chromosome aberrations (Figure 1D); this suggests that the breaks arising as a consequence of collapsed replications forks is not the only mechanism by which 5-azadC induces DNA damage. Indeed, transcription inhibition abrogates the levels of 5-azadC induced chromosome aberrations (Figure 1D). Also, we observed that this abrogation was not caused by a reduction in the mitotic index (Supplementary Figure 1A) or by variations in cell cycle profiles (Supplementary Figure 1B and 1C). Furthermore, our experimental schedule is similar to other previously published in relation with Topoisomerase I adducts [23].

### A transcription dependent DNA damage response (TC-DDR) is activated after 5-azadC incorporation

It has been shown that Topoisomerase I-DNA complexes can block transcription forks resulting in the activation of a DDR involving ATM-CHK2, γ-H2AX, 53BP1 and DNA-PK [24, 25]. H2AX is phosphorylated at Serine 139 in response to several types of DNA damage such as DNA DSBs [26] and replication stress [27]. 53BP1 foci are a more restrictive marker for DSBs and are thought to be indicative of a NHEJ repair [28, 29].

**Figure 2:**
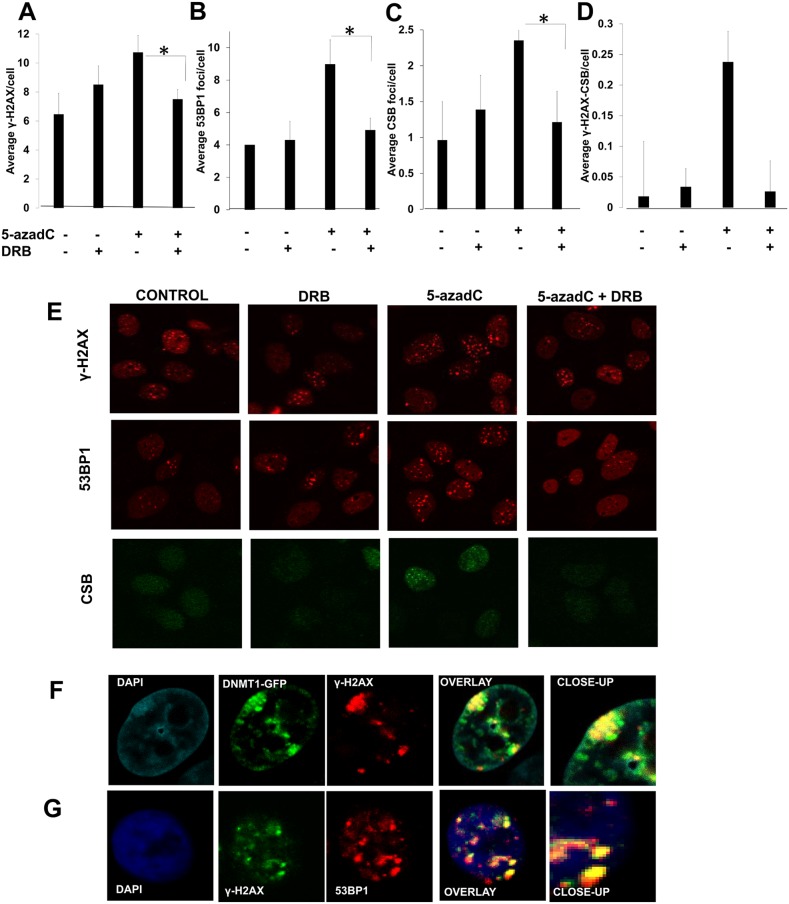
A transcription dependent DNA damage response is activated after 5-azadC incorporation CS1AN CSB-GFP complemented cells were treated following the schedule presented in Figure 1A and γ-H2AX **(A)**, 53BP1 **(B)** and CSB **(C)** foci were analyzed. The average of γ-H2AX foci colocalizing with CSB per cell is representated in **(D)**. Representative micrographs are depicted in **(E)**. **(F)** Co-localization between DNMT1 and γ-H2AX foci. AA8 cells were transfected with DNMT1-GFP plasmid, treated for 6 h with 5-azadC and immunomarked for γ-H2AX. **(G)** Co-localization between 53BP1 and γ-H2AX. AA8 cells were treated for 6 h with 5-azadC and cells were fixed and marked for immunofluorescence. Confocal micrographs are depicted. The means and SEM of two-three independent experiments are shown. Statistical significance was determined using Student's *t*-test, n.s. = non-significant, ^**^P < 0.01, ^*^P < 0.05.

Using the same experimental design described above (Figure 1A), we wanted to analyze if a TC-DDR is activated. We used human cells defective in CSB from a Cockayne syndrome patient complemented with human CSB (CS1AN CSB-GFP). According to the results presented before, 5-azadC induced an increase in the phosphorylation of H2AX which was reduced when cells were co-treated with DRB (Figure 2A). Similar results were found when examining 53BP1 foci (Figure 2B).

CSB has been recently proposed to have a role in the repair of Topoisomerase I cleavable complexes stabilized by Camptothecin (CPT) [30]. Under our experimental design we found an accumulation of CSB in foci after a treatment with 5-azadC that seems to be transcription dependent (Figure 2C).

We found that γ-H2AX co-localized with CSB foci, and that the frequency of 5-azadC induced co-localized foci was reduced when transcription was inhibited (Figure 2D).

When looking at co-localization in DNMT1-GFP transfected cells, we found that DNMT1 foci co-localized with γ-H2AX. This data indicates the activation of a DDR when 5-azadC was administered at short times without the interference of a second replication round (Figure 2E). Under these conditions, γ-H2AX also co-localized with 53BP1 foci (Figure 2F). All these data suggest that transcription has a role in the sensing of the lesions induced by 5-azadC.

### CSB mutant cells have defects in the signaling of DNA damage induced by 5-azadC

**Figure 3:**
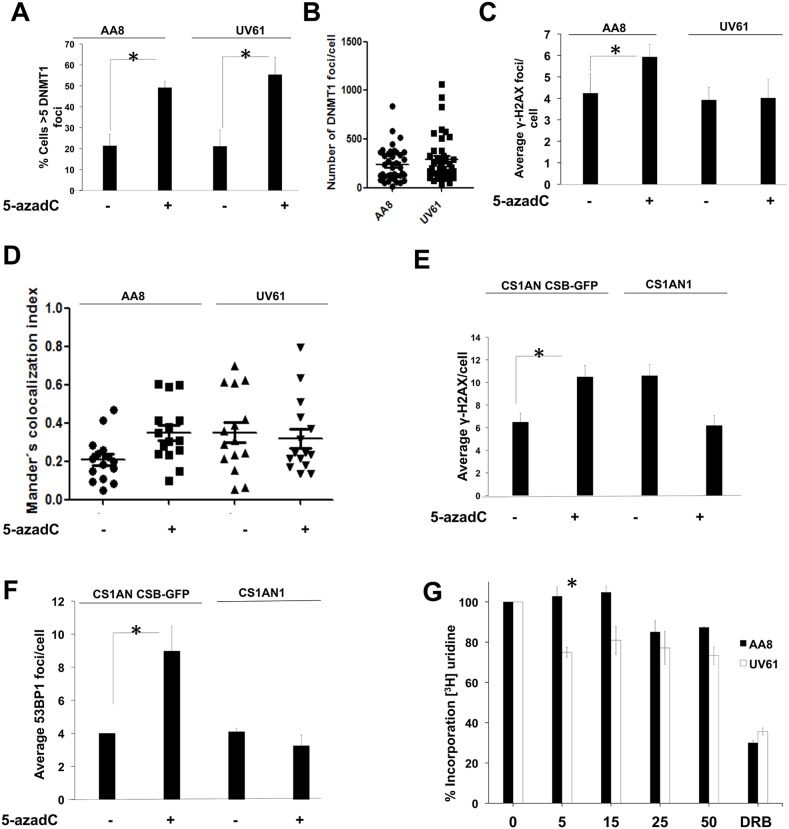
CSB mutant cells have defects in the signaling of the initial DNA damage induced by 5-azadC **(A-B)** Similar levels of trapped DNMT1 in wild type AA8 and CSB defective UV61 cells. DNMT1 CSB-GFP cells were treated for 6 h with 15μM of 5-azadC, fixed and prepared for scoring of DNMT1 foci. Cells > 5 foci were scored as positive (A). Alternatively the number of DNMT1 foci/cell is also represented (B). **(C)** CSB cells have defects forming γ-H2AX foci in response to a short 5-azadC treatment (6 h). **(D)** 5-azadC induces DNMT1-γH2AX co-localization in a CSB dependent fashion. DNMT1-GFP transfected cells were treated for 6 h with 5-azadC, fixed and immunolabelled for γH2AX. Mander's co-localization index was employed (Image J). **(E and F)** Importance of CSB in the formation of γ-H2AX and 53BP1 foci in human cells. CS1AN and CS1AN CSB-GFP cells were treated for 6 h with 5-azadC (15μM), fixed and immunolabelled for the indicated proteins. A clear increase in foci was observed for complemented cells. However CSB cells couldn't form repair foci. **(G)** CSB cells present transcription blocks after a short treatment with 5-azadC. AA8 and UV61 cells were treated with increasing doses of 5-azadC for 6 h and labelled with tritiated uridine for 15 minutes in order to monitor transcription rates. CSB cells presented blocks in transcription elongation from the first dose assayed. However for wild type cells blocks are evident only for higher doses assayed.

Once described that the initial DNA lesions induced by 5-azadC are sensed in a transcription dependent fashion, we wanted to address the importance of CSB in the setup of this process. We use UV61 and CS1AN cells (CSB deficient) and data were compared with AA8 (wild type) or CS1AN CSB-GFP (CSB complemented) cells. Cells were treated for 6 hour with 5-azadC and the frequency of DNMT1 foci was monitored in wild type and CSB deficient cells. Our results showed no difference in the number of DNMT1 foci in both cell lines after 5-azadC treatment (Figure 3A–3B). Therefore, the frequency of initial lesions seems to be similar, which makes possible to compare the DDR activated in both cell lines. We found that UV61 cells were unable to phosphorylate H2AX in response to 5-azadC (Figure 3C). Accordingly, we could not find an increase in the co-localization between γ-H2AX and DNMT1 in response to 5-azadC in CSB mutant cells (Figure 3D).

When we wanted to analyse γ-H2AX and 53BP1 foci in human cells, a similar pattern was found, which demonstrates a similar behaviour of hamster and human cells (Figure 3E–3F).

A classical feature of CSB-deficient cells is related with its transcriptional incapability to recover from a transcription block [8]. In order to monitor the effect of 5-azadC in the transcription rates, we incubated AA8 and UV61 cells with increasing doses of 5-azadC for 6 h. Then, cells were pulse-labelled for 15 minutes with ^3^H-Uridine and transcription rates were measured in a scintillation counter. Our data shows a consistent decrease in transcription rates in UV61 cells from the first dose assayed (5μM). However, the block started to be evident only when high doses of the drug were assayed for wild type cells (Figure 3G). Altogether, our results show that CSB is involved in the signaling of 5-azadC lesions required to resume transcription.

### Delayed repair of trapped DNMT1 in CSB mutant cells

Trapped DNMTs are the primary lesion induced by 5-azadC responsible for tumour cell death [31]. It has been recently shown that trapped Topoisomerases by poisons are obstacles to transcription, causing blocks that act as sensors for the repair of the adducts and the resumption of transcription [24]. Moreover CSB has been recently implicated in this mechanism [30].

**Figure 4:**
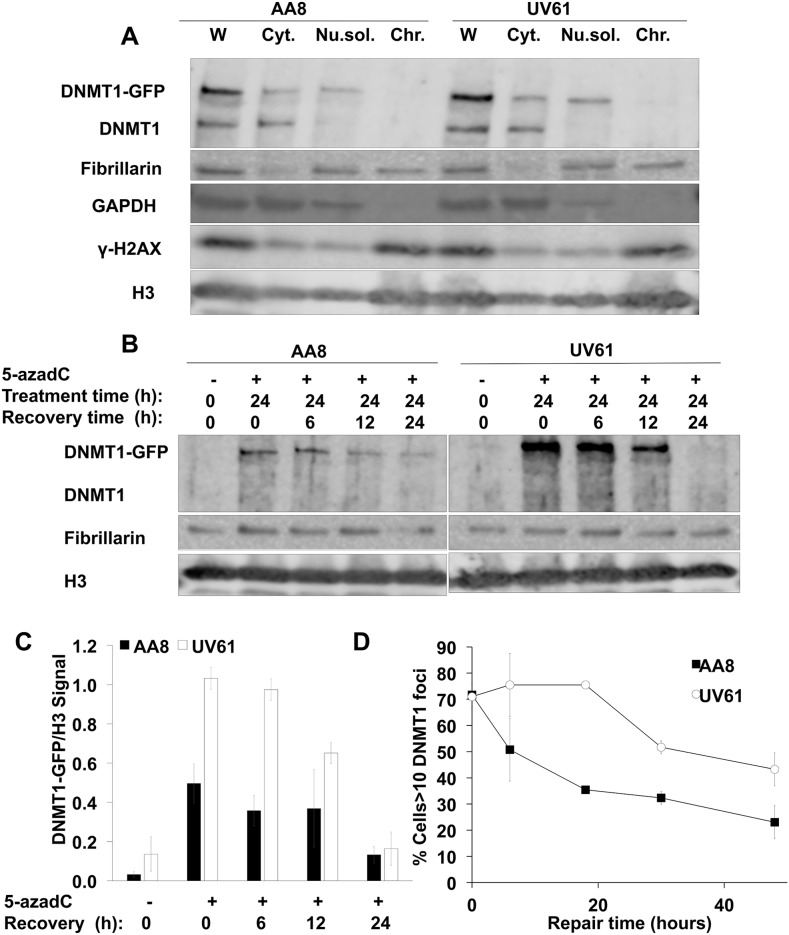
Delayed repair of trapped DNMT1 in CSB mutant cells **(A)** Validation of the fractionation protocol followed. AA8 and UV61 cells were transfected with DNMT1-GFP plasmid before lysis. Whole (W) or fractionated lysis were carried out. Cytoplasmic (Cyt), nuclear soluble (Nu.sol.) and chromatin (Chr.) fractions were obtained. Our data show that endogenous and ectopic DNMT1 are equally expressed in both cell types. The expected localization of GADPH, Fibrillarin and γ-H2AX in the cytosolic, nuclear, and chromatin enriched fractions, respectively is shown. **(B)** Repair of chromatin bound DNMT1-GFP in wild type AA8 and CSB deficient UV61 cells. Cells were treated for 24h with 15μM of 5-azadC and allowed to repair in drug free media for the indicated times. Cells were fractionated and chromatin fractions were loaded analyzed by SDS-PAGE. **(C)** A densitometric analysis of the chromatin bound DNMT1-GFP in relation to the chromatin marker H3 is shown. **(D)** Alternatively DNMT1-GFP transfected AA8 and UV61 cells were treated for 24h with 15μM of 5-azadC and allowed to repair in drug free media for different times, fixed and scored for DNMT1 foci in a fluorescence microscope. The means and SEM of two independent experiments are shown. Data were considered statistically different when P < 0.05 (^*^) or P < 0.01(^**^).

Based on the evidence reported above, our data suggests an implication of CSB in the signaling of trapped DNMT1. If this is the case, it is conceivable to think that CSB deficient cells would be defective in the repair of trapped DNMT1 induced by 5-azadC. To address this question, we performed cellular fractionation to isolate chromatin in which we detected trapped DNMT1. We show that DNMT1-GFP transfected AA8 and UV61 cells presented similar levels of native and ectopic DNMT1 in the whole lysates (Figure 4A).

In order to monitor the repair of trapped DNMT1, DNMT1-GFP transfected cells were treated with 15μM of 5-azadC for 24 h, washed and allowed to recover for different times in drug free media. When looking at the levels of remaining DNMT1-GFP trapped in chromatin, we found a clear increase in UV61 cells compared to wild type cells (Figure 4B). Taking into account that there is some repair during the treatment time, a plausible explanation would be a defect in the repair in CSB deficient cells, responsible for the increased level of trapped DNMT1 observed after treatment. Also, we think that this repair defect in CSB mutant cells would be the cause for the delayed repair observed as well.

This result was confirmed by fluorescence microscope in DNMT1-GFP transfected cells (Figure 4D). In this case, DNMT1 foci were scored over time after a 24 hour treatment with 5-azadC. Our data show that CSB cells present more DNMT1 foci all along the repair time course. These findings agree very well with data presented above and highlight the importance that CSB has in the repair of trapped DNMT1.

### Increased levels of DNA damage and reduced survival in CSB mutant cells after 5-azadC treatment

**Figure 5:**
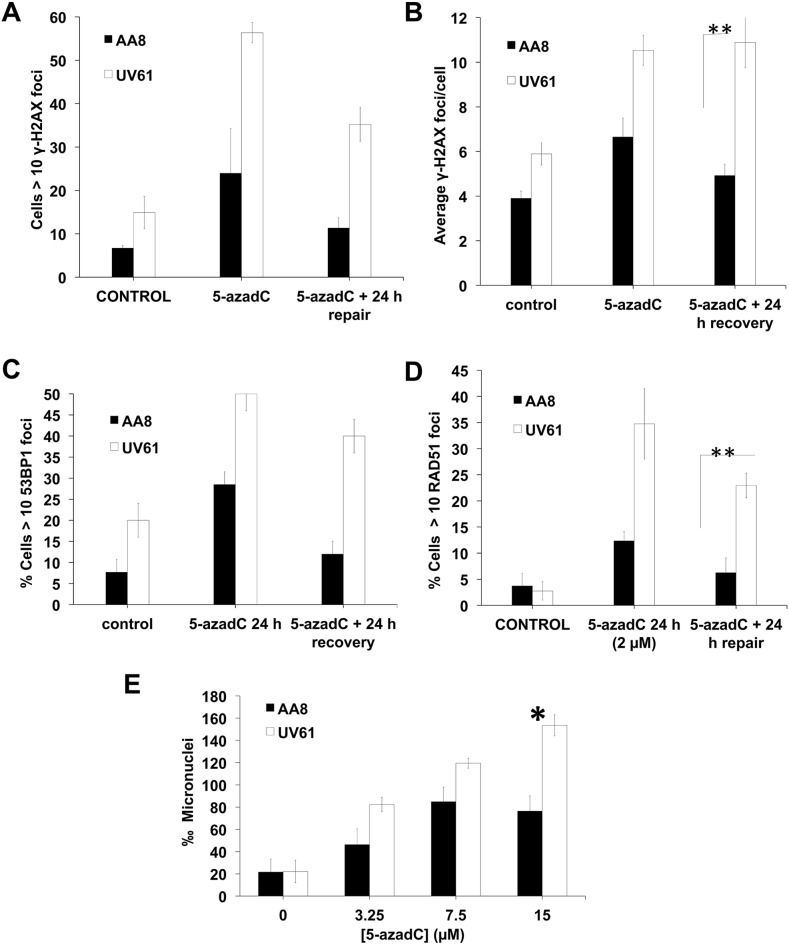
Increased levels of DNA damage in CSB mutant cells after a prolongued 5-azadC treatment **(A–D)** AA8 and UV61 cells were treated for 24 h with 5-azadC (2μM) washed and allowed to repair for 24h. Then were fixed and prepared for immunological staining of γ-H2AX (A-B), 53BP1 (C) and RAD51 (D). **(E)** In order to monitor residual DNA damage after a 24h treatment with 5-azadC, we performed micronucleus assay. Cells were treated with increasing doses of 5-azadC, washed and allowed to repair in cytochalasin B containing media for 18h. Cells were then fixed and scored for the number of micronuclei in binucleated cells. The means and SEM of three independent experiments are shown. Data were statistically analysed using Student's *t*-test. Data were considered statistically different when P < 0.05 (^*^) or P < 0.01(^**^).

Recently, we have shown that trapped DNMTs interfere with replication and generate DNA damage [21]. If CSB mutant cells have defects in the repair of trapped DNMT1, the levels of DNA damage should increase over time. To test this hypothesis, we treated cells with 5-azadC for 24 h and allowed to repair for another 24 h. DNA damage was evaluated by the scoring of γ-H2AX, 53BP1 and RAD51 foci (Figure 5A–5D). We found that, in contrast to the data obtained when we used short treatments (Figure 2), CSB mutant cells display increased levels of DNA damage foci. In our opinion this apparent contradiction can be explained considering that foci arising after short treatments indicate initial signaling of the repair of 5-azadC lesions. On the contrary, many of the foci visualized after long treatments could be related with the signaling of secondary lesions i.e. collisions with the replication fork with trapped DNMT1.

On the other hand, CSB deficient cells showed a high increase in 5-azadC induced RAD51 foci as compared to wild type cells (Figure 5D). We hypothesize that CSB cells could present increased levels of collapsed forks as a result of unrepaired DNMT1 triggering RAD51-dependent HR for repair.

Of interest, CSB deficient cells showed increased micronuclei frequency, which is indicative of residual DNA breaks (Figure 5E).

**Figure 6:**
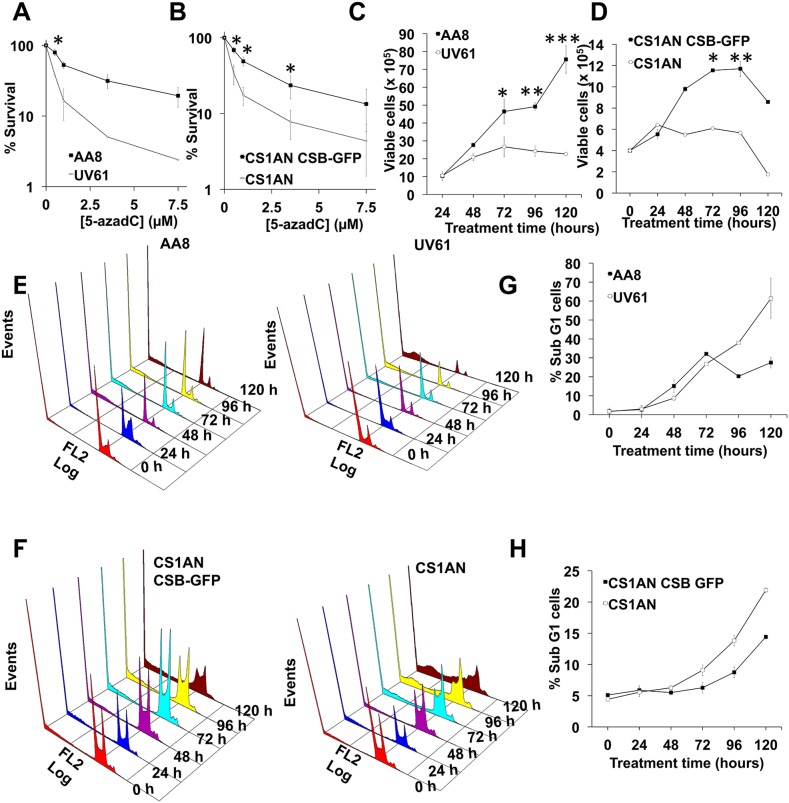
CSB promotes survival after 5-azadC incorporation **(A and B)** Survival after 5-azadC treatments. AA8 and UV61 cells (A) or CS1AN and CS1AN CSB-GFP cells (B) were seeded at low density, treated with 5-azadC for 24h and allowed to repair in fresh media for 7-9 days to form colonies. Colonies were fixed and stained with methylene blue/methanol 4 g/L. Colonies with more than 50 cells were scored. Data are plotted in relation to the survival of control cells (100 %). **(C and D)** Cell growth after a continuous exposure to 5-azadC (15μM). Cells were collected at the indicated times and viable cells were scored (tripan blue negative). **(E–H)** Cell cycle profiles after a continuous exposure to 5-azadC (15μM) in AA8-UV61 (E), CS1AN-CS1AN CSB-GFP (F) and quantifications of subG1 cells (G-H). The means and SEM of three independent experiments are shown. Data were statistically analized using Student's *t*-test. Data were considered statistically different when P < 0.05 (^*^) or P < 0.01(^**^).

Next we wanted to examine if DNA repair defects observed in CSB mutant cells have a negative consequence in survival. We performed clonogenic assays both in hamster and human cells (Figure 6A–6B). As can be seen, CSB mutant cells (UV61 and CS1AN) presented reduced survival when exposed to 5-azadC. In good agreement with this, CSB mutant cells also showed a reduced growth when they were chronically exposed to 5-azadC (Figure 6C–6D). Cell cycle profiles agree very well with the above data and showed increased levels of cell death (sub-G1 cells) in CSB mutant cells when treated with 5-azadC (Figure 6E–6H).

### Transcription and CSB work in the same pathway to promote survival after treatment with 5-azadC

**Figure 7:**
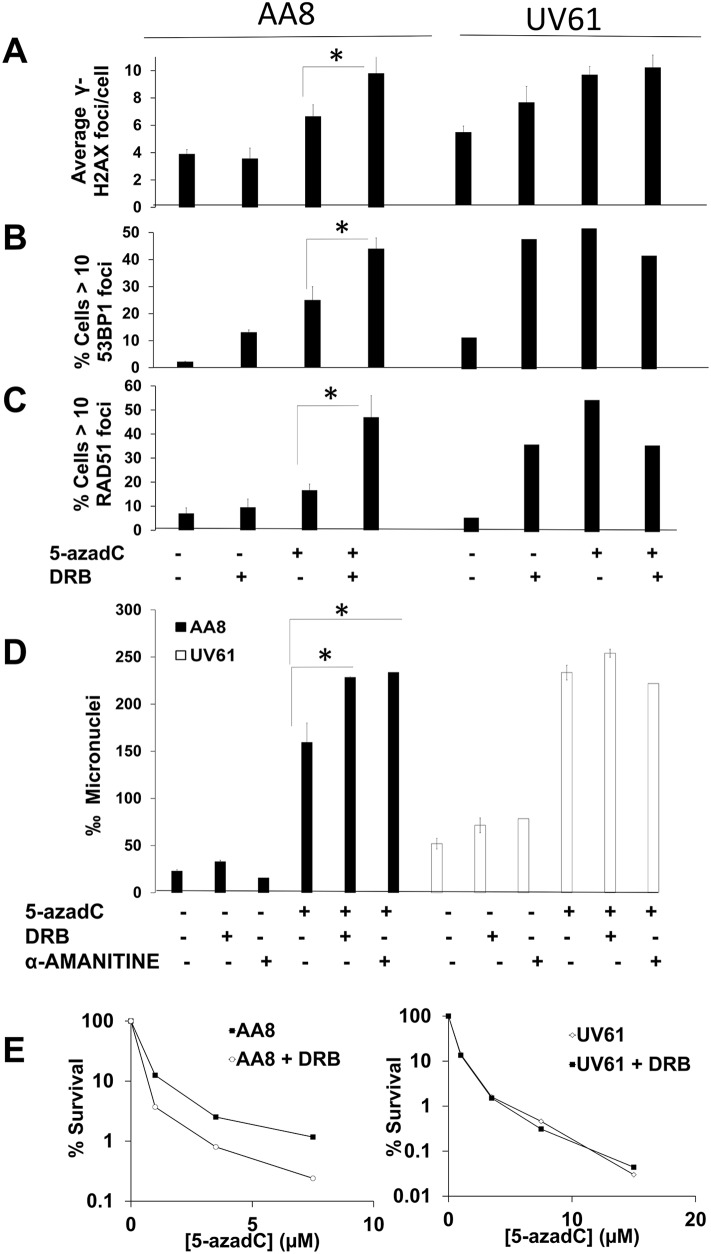
Prolonged transcription inhibition potentiates 5-azadC-induced DNA damage in a CSB dependent manner **(A–C)** Cells were treated for 24h with 5-azadC (2μM), DRB (10μM) was added in the last 12h. Cells were then fixed and immunomarked for γ-H2AX (A), 53BP1 (B) and RAD51 (C). **(D)** DNA damage was also scored with the micronucleus test. Exponential cell cultures were treated 5-azadC ( 15μM) alone or in combination with DRB (20μM) or α-Amanitin (1μM) and allowed to recover in cytochalasin B containing media for 18h, fixed and the frequency of micronucleus was scored in binucleated cells. The means and SEM of three independent experiments are shown. Data were statistically analyzed using Student's *t*-test. Data were considered statistically different when P < 0.05 (^*^) or P < 0.01(^**^). **(E)** Transcription inhibition sensitizes wild type cells but not CSB deficient cells to 5-azadC. AA8 and UV61 cells were seeded at low density and 4h later treated for 36h with increasing doses of 5-azadC. Last 24h cells were in the presence of DRB (20μM). Cells were then washed and allowed to form colonies in drug free media for 7-9 days. Colonies were then fixed and stained with methylene blue in methanol (4g/L). Data are plotted as percentage of survival in relation to control cells (100 %). A representative experiment is shown.

Apart from TC-NER, CSB has been implicated in several processes such as Base Excision Repair [11, 32, 33], nucleosome positioning [34] and mitochondrial DNA repair [35]. As far as we know, many of these processes are not closely related to transcription elongation. Therefore, we analyzed if the function of CSB is epistatic with transcription elongation in response to 5-azadC. We treated AA8 and UV61 cells with 5-azadC for 24 h. During the last 12 h cells were cultured in the presence/absence of the transcription inhibitors DRB or α-Amanitin. Next, we monitored DNA repair foci (Figure 7A–7C) or micronuclei (Figure 7D) induction. An increase of DNA repair foci induced by 5-azadC was observed when transcription was blocked in wild type cells but not in UV61 cells (Figure 7A–7C). The same pattern was observed when looking at micronuclei induction (Figure 7D). The lack of potentiation in micronuclei induction in CSB deficient cells cannot be explained by a delay in cell cycle, as no difference in the progression of the cell cycle was observed (Supplementary Figure 2). Overall these results suggest that transcription works in the same pathway than CSB to repair lesions induced by 5-azadC.

Finally, we wanted to see if the epistatic interaction could be also observed when looking at cell survival (Figure 7E). In good agreement with the data presented above, a clear sensitization by DRB was obtained just in wild type cells. As a whole, data suggest that transcription works upstream to CSB in response to lesions induced by 5-azadC.

## DISCUSSION

Several DNA damaging agents are known to form DNA-protein cross-links (DPC), including topoisomerase I and II poisons, 5-azadC, PARP inhibitors and cisplatin. In recent years, some mechanisms involved in the repair of these DNA insults have been elucidated [36], although many questions remains unanswered for the DPC induced by 5-azadC.

Immobilized proteins onto DNA are described to be very effective disruptors of transcription [37], and are preferentially repaired if they are in the transcribed strand [38]. The arrest of RNApol II at transcription blocking lesions stops the elongation of the newly synthesized RNA and if the block is prolonged in time, leads into apoptosis.

It has been shown that transcription blocks trigger a different ATM-dependent DNA damage response named non-canonical ATM activation [24, 25, 39]. In this paper we designed a 6 hour schedule to monitor the initial events in the repair of trapped DNMT1 by 5-azadC. Under these conditions we assume that the canonical ATM response that arises when replication forks are collapsed with trapped DNMT1 is not activated [40].

Firstly we demonstrate that a short treatment with 5-azadC induces either DNA breaks (Figure 1) and repair foci (Figure 2) in a transcription dependent manner. As far as we know this is the first demonstration that a TC-DDR is activated soon after trapping of DNMT1. These repair foci including γ-H2AX and 53BP1 depends on functional CSB to be established, as no induction of foci was found when CSB deficient cells were treated for a short time with 5-azadC (Figure 3A–3F). Furthermore, we demonstrate that the treatment with 5-azadC for a short time, decreases the transcription rates of CSB mutant cells (Figure 3G), which suggests that transcription in mutant cells cannot be resumed after 5-azadC treatment. Similarly, a lack of 53BP1 and γ-H2AX foci induction was found when CSB deficient cells were treated for a short time with CPT [41] or Ecteinascidin 743 [42] respectively. Both drugs are well known inductors of transcription coupled DNA damage. However, there is another recent contribution where it is shown that the levels of 53BP1 foci are increased in CSB deficient cells soon after a ionizing radiation exposure [15]. A plausible explanation to this controversy could be the different implications of TC-DDR in relation with the agents studied. It is likely that the role of CSB when recruited to IR-induced DSBs differs from its implication in bulky lesions such as those induced by Ecteinascidin 743, Topoisomerase I poisons or 5-azadC.

When RNApol II is stalled at damage sites, a CSB-dependent recruitment of CSA/E3 ubiquitin ligase complex is followed by the recruitment of other NER factors [43]. However, classic NER proteins do not seem to be important in survival after 5-azadC exposure, so the repair mechanism behind DNMTs-DNA adducts is not a classical TC-NER (see reference [44] and Supplementary Figure 3).

We also show an increase in the colocalization levels between DNMT1 and γ-H2AX when exposed for a short time to 5-azadC in wild type cells, which indicates that the activated DDR is related to trapped DNMT1 (Figure 2F and 3D). In support of this notion, we show a delayed repair of trapped DNMT1 in CSB deficient cells (Figure 4). Future work is needed to elucidate the exact mechanism of this repair; however it is interesting to mention that CSB contains a PARP binding site and that there is epistatic evidence that PARP1 and CSB act together in repair of oxidative lesions [45]. Interestingly, we have reported that the PARP inhibitor Olaparib delays the clearance of DNMT1 foci after 5-azadC [22]; we can speculate that PARP and CSB are working in an epistatic fashion to repair these DNA lesions.

Apart from protein-DNA adducts, 5-azadC also induces base damage that can be converted into single strand breaks (SSB) after BER processing [22] and SSB can also be a strong block to transcription [46]. As 5-azadC is incorporated all over the genome but concentrated in CpG islands, it is possible that clustered lesions (comprised by SSB and trapped DNMTs) can be formed in those islands and transcription could be stopped by both types of lesions.

DNA methylation is key epigenetic mechanisms consisting on the covalent addition of a methyl group to the carbon at position 5 of the cytosine in the CpG dinucleotides. Around 80% of methylation occurs at CpG sites in mammalian genomes [47]. These palindromic dinucleotides are concentrated in regions called CpG islands, that can be found both in gene promoters as well as in gene bodies, where they can regulate splicing [48] and expression of noncoding RNAs [49]. Single CpGs are often found in repetitive DNA elements and centromeric regions that are not related to transcription.

During replication, methylation pattern is maintained by DNMT1 [50]. This 180 kDa enzyme methylates hemimethylated DNA acting just after replication forks. 5-azadC is randomly incorporated into DNA instead of normal cytosine. Therefore, DNMTs could be trapped either in islands surrounding the transcription start site as well as in intragenic islands. We think that the transcription fork could be blocked with the incorporation of DNMT1-DNA adducts in those places triggering a DDR.

When 5-azadC is administered for prolonged periods (24 h), we found higher levels of DNA repair foci in CSB deficient cells (Figure 5). Increased levels of 53BP1 and RIF1, as well as reduced levels of RAD51 foci, have been found in CSB deficient cells treated with ionizing radiation. These data support an active role of CSB in the DSB repair pathway choice: facilitating HR and suppressing Non-Homologous-End-Joining (NHEJ) [15]. After a 5-azadC treatment, we found higher levels of γ-H2AX and 53BP1 foci, as well as increased levels of RAD51 foci, which suggests that HR is not impaired in our CSB deficient cells. We believe that foci arising after long 5-azadC treatments differ from those observed when 5-azadC is present only for 6 h. We hypothesize that a short treatment schedule reveals only the initial DNA damage repair, i.e., transcription coupled repair of trapped DNMTs. However, when long treatment schedules were assayed, both repair of trapped DNMTs and its collisions with replications forks (probably occurring at the same time) are visualized. The reduced levels of the initial repair foci in CSB deficient cells are explained by a possible role of CSB in a DDR response after transcription blocks. If CSB is not functional, then more unrepaired lesions are present in DNA which can interfere with second round replication forks, leading to more DBSs with time.

**Figure 8:**
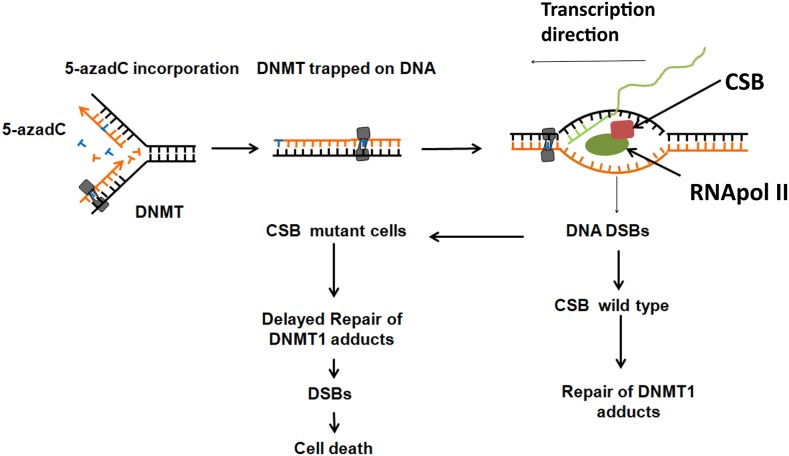
Proposed model of CSB in the toxicity of 5-azadC 5-azadC is randomly incorporated instead of normal cytosine during DNA replication and attempted to be methylated at inter- and intragenic CpG islands by DNMT1 which remain trapped generating protein-DNA adducts. Transcription forks are blocked at these adducts generating DNA DSBs that need to be properly signaled in a CSB dependent manner. CSB proficient cells are able to repair the adducts but not CSB deficient cells. If these adducts are not repaired, they could interfere with the progression of the replication forks of the following cell cycle and generate DSBs and cell death.

All data compiled in this paper can be summarized as follows (Figure 8). While replication forks advance, DNMT1 methylates cytosines present in CpG sites. When 5-azadC is in the place of normal cytosine, DNMT1 remains trapped generating DNA damage. These bulky lesions block transcription forks and generate DNA breaks. According to our data, CSB orchestrates a DDR of these lesions. If cells have functional CSB, then a proper DNA damage response is activated and DNA repair proteins are recruited; this allows repair and resumption of transcription. In the absence of CSB, DNA adducts are not repaired and could represent an obstacle to next replication and as a consequence DNA breaks and cell death.

## MATERIALS AND METHODS

### Cell culture and chemicals

Chinese hamster ovary cells AA8 (wild-type) and UV61 (CSB deficient) were a gift from Larry Thompson and were cultured in DMEM media. The human CSB (ERCC6) gene corrects the transcription coupled repair defect in UV61 cells [51].

CS1AN cells are SV40 immortalized patient fibroblasts deficient in CSB and were a gift from Jurgen Marteijn (Erasmus MC, Department of Genetics, Wytemaweg 80 3015 CN Rotterdam). These cells were complemented with CSB-GFP [52].

All cell lines were cultured with 10% fetal bovine serum, penicillin (50 U*/*ml) and streptomycin (50 μg*/*ml), at 37°C in a 5% CO2 atmosphere.

5-aza-2′-deoxycytidine (Sigma) was dissolved in phosphate buffered saline (PBS), aliquoted and stored at −80°C. 5,6-Dichlorobenzimidazole 1-β-D-ribofuranoside (DRB), α-Amanitin and Cytochalasin B (Cyt B) were obtained from Sigma, dissolved in DMSO, aliquoted and stored at −80°C. DRB inhibits CK2 kinase which phosphorylates and activates RNA polymerase II, resulting in repression of transcription elongation [53] and dramatic reduction in mRNA levels [54].

### Immunofluorescence

Exponential growing cells were treated for 24 h with 5-azadC. During the last 12 h of this treatment cultures were grown in the presence or absence of DRB (10μM).

Alternatively, cells were treated with 5-azadC (15μM) for 6 h and DRB (30μM) was present during the last 3 h.

After treatments, cells were washed in PBS and incubated for 30s with ice cold 0.1% PBS containing Triton-X in order to pre-extract soluble protein. Then cells were fixed in 4% paraformaldehyde at room temperature for 10 min. Immunofluorescence of γ-H2AX (millipore), 53BP1 (Santa Cruz) and RAD51 (Santa Cruz) were carried out as previously described (21). Cells were photographed using a Zeiss LSM 7 duo confocal microscope.

The number of foci per cell was assessed using Image J software (NIH). Also, the percentage of cells with more than 10 or 20 foci were scored. At least 200 nuclei were counted on each slide.

### Plasmids and transfection procedures

The plasmid expressing GFP-tagged DNMT1 was a gift from Keith Robertson and has been described previously [20].

All transient transfections were performed using JetPRIME^®^ transfection reagent according to the manufacturer's protocol (PolyPlus). Cells were allowed to express DNMT1-GFP for 24 h, cells were then treated with 5-azadC (15μM) for 6 h and fixed using 4% paraformaldehyde. Samples were then processed for the detection of γ-H2AX foci according to previous papers (21). Manders colocalization index was measured using Fiji software (Coloc2 plugin, where 1 means perfect co-localization and 0 no co-localization).

For repair experiments, 24 h after DNMT1-GFP transfection, cells were trypsinized reseeded and treated with 5-azadC (15μM) for 24 h. Media was then replaced and cells were fixed at different times using paraformaldehyde 4%. The percentage of cells with > 10 DNMT1 foci in transfected cells was scored [22].

### Survival assay

Cells were seeded at low density in 10 cm Petri dishes 4h prior to 5-azadC treatment for 24h. Media was then replaced and colonies were allowed to grow for 8–10 days.

For combination experiments cells were treated for 12 h with increasing doses of 5-azadC. Then DRB (20μM) was added to the cultures for 24 h. At this point media was replaced and colonies were allowed to grow for 8-10 days.

Colonies were then fixed and stained (methylene blue dissolved in methanol, 4g*/*l). The data are plotted as percentage of survival referred to control cells.

### Micronucleus assay

Cells were seeded and allowed to grow for 24 h. Next day, cultures were treated with increasing doses of 5-azadC for another 24 h. For combined experiments, cells were treated for 12 h with 5-azadC, media was then replaced and cells were allowed to grow in the presence of the transcription inhibitors α-Amanitin (1μM) or DRB (20μM) for 12 h.

Finally, for all experiments, media was changed and cultures were treated with 3 μg/ml of Cytochalasin B for 18 h. Cells were trypsinized and fixed in methanol-acetic solution (3:1). Preparations were made by dropping cells onto wet slides and staining with Giemsa (3% in Sörensen buffer). Two thousand binucleated cells were scored for micronucleus frequency in each treatment.

### Chromosome analysis

Exponential growing AA8 and UV61 cells were cultured for 6 h with 5-azadC (50μM). DRB (30μM) was added during the last 3 h. Then mitotic arrest was achieved with 2×10^−7^ M of colcemid for 2.5h.

Cells were collected and incubated in hypotonic solution (0.075M KCl) for 2 min, fixed in methanol: acetic acid (3:1) and dropped onto microscope slides. Slides were stained with 3% Giemsa/Sörensen's buffer for 5 min and mounted in D.P.X. (Sigma).

Two hundred complete metaphases were evaluated in each experimental point. Images were taken with a Nikon eclipse 50i microscope equipped with a Nikon DS-Fi1 camera and using the 100-fold magnification objective (numerical aperture 1.25) and the NIS Elements 3.0 adquisition software (Nikon).

### Cellular fractionation and SDS-PAGE analysis

In order to measure the trapping and repair of DNMT1 in DNA, we performed cellular fractionation as described before [22, 55]. After treatment, cells were collected at different time points and counted separately using a Bio-Rad TC20™ cell counter. In order to load equal amounts of chromatin fractioned protein, 1×10^6^ cells were fractioned for each sample. All protein were separated on sodium dodecylsulphate-polyacrylamide gel electrophoresis (SDS-PAGE) gels and subsequently transferred to nitrocellulose membranes. All subsequent steps were carried out in TBS–Tween (10 mM Tris-HCl, pH7.6, 150mM NaCl and 0.05% Tween-20) either containing 5% milk (blocking and antibody incubations) or 5% BSA (phospho-specific antibody incubations). Antibody binding was visualized by enhanced chemiluminescence using the SuperSignal West Dura or Pico reagents from Pierce™. Antibodies used: DNMT1 (Abcam), Histone H3 (Santa Cruz), GAPDH (Abcam) and Fibrilarin (Santa Cruz).

### Transcription assay

In order to measure transcription rates we seeded 50.000 AA8/UV61 cells in 24 well plates. After 24h, they were treated with increasing doses of 5-azadC. 6 h later, 1μCi of [^3^H]-Uridine was added for 15 minutes at 37°C, washed once with PBS and ice cold-methanol extracted for 30 minutes. Cells were washed again with PBS and 500μl of 0.01M NaPO_4_H_3_ in 0.5% SDS buffer was added. Cells were then dislodged with a cell scraper, transferred to 1.5 ml tubes and sonicated in water bath for 10 minutes. Finally lysates were transferred to scintillation tubes and 3ml of Ultima Gold scintillation fluid were added before analysis.

### Flow cytometry

Exponential cells were treated with 5-azadC (15 μM) and collected at different time points. Then cells were counted and cell viability was monitored using trypan blue exclusion assay. Cells were washed in ice-cold PBS and fixed in 70% ethanol for 24h at −20°C. Subsequently, cells were washed and incubated with 500μl of a solution containing Propidium iodide (0.1 mg/ml) and RNAse (8 μg/ml) for 1.5 h at 4°C. Cell cycle profiles were obtained in a Beckman Coulter Flow cytome ter model: CYTOMICS FC500-MPL.

## SUPPLEMENTARY MATERIALS FIGURES AND TABLES


