# Supplementary material for: The Cockayne syndrome protein B is involved in the repair of 5-AZA-2′-deoxycytidine-induced DNA lesions
Source: Oncotarget. 2018 Oct 12;9(80):35069–84. doi: 10.18632/oncotarget.26189 (PMC6205548; doi:10.18632/oncotarget.26189)
Supplement: Supplementary file 1 [file oncotarget-09-35069-s001.pdf]

The Cockayne syndrome protein B is involved in the repair of 5-AZA-2’-deoxycytidine-induced DNA lesions

SUPPLEMENTARY MATERIALS

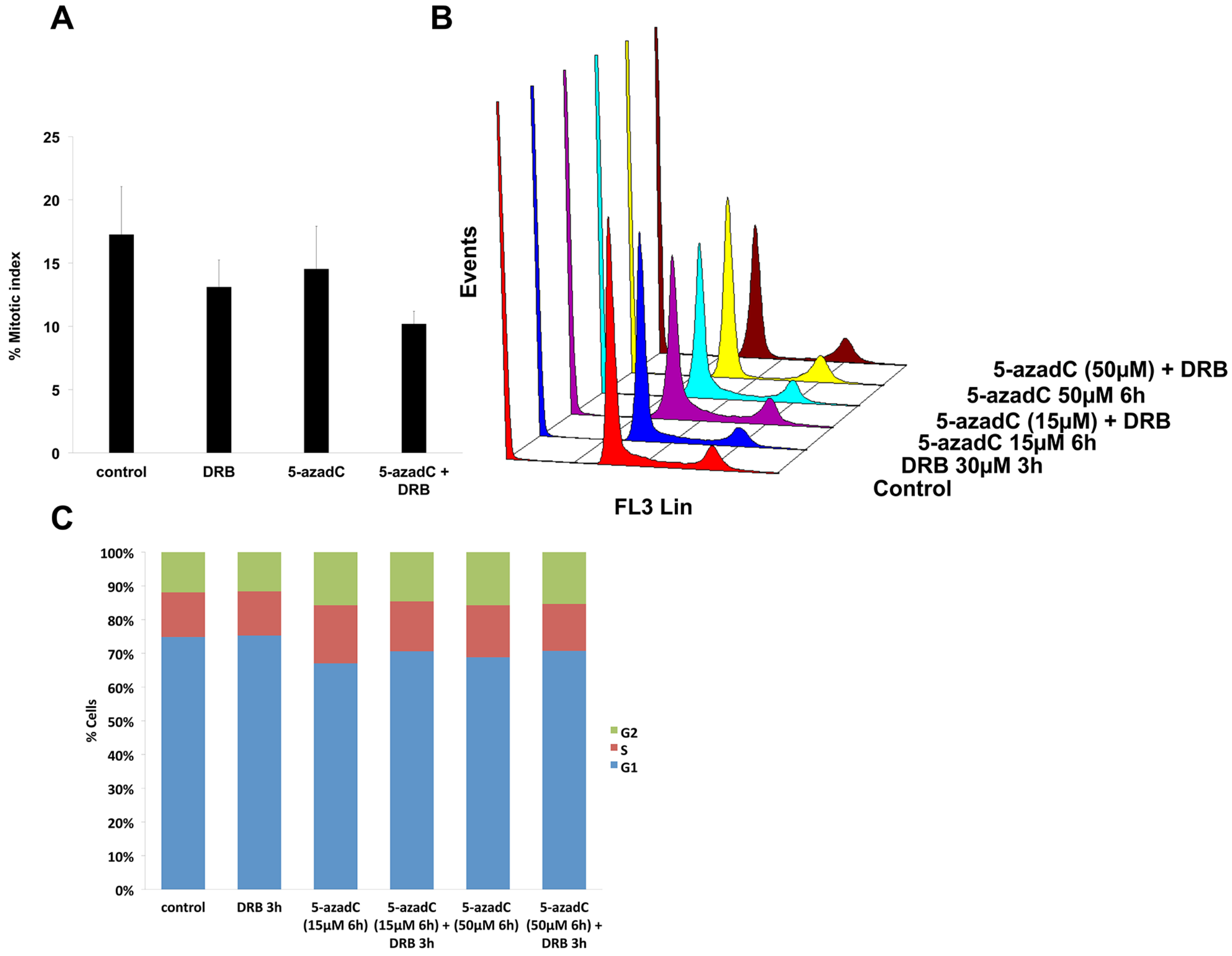

Supplementary Figure 1: (A) Mitotic index of AA8 cells exposed to 5-azadC. Exponential AA8 cells were treated with 50µM of 5-azadC for 6h. Last 3h cells were treated with DRB (30µM). Cells were then treated with colcemid for 2.5h, trypsinized and fixed. Cells were spread onto glass slides and stained with Giemsa 3% in Sörensen buffer. We depict average and SEM from three independent experiments. *t*-test revealed no statistically significance (P=0.19) between 5-azadC and co-treated cells. **(B)** Cell cycle profiles after a 6 hour treatment with 5-azadC in combination with DRB. At the end of the treatments, cells were processed according to the material and methods section. **(C)** Quantification of the cell cycle histograms described in B.

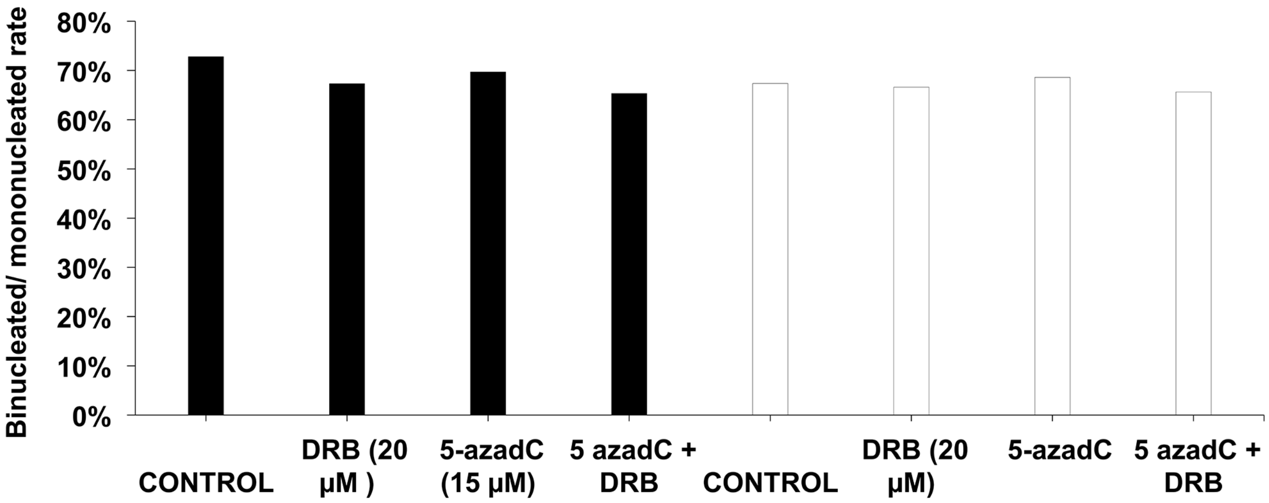

Supplementary Figure 2: Cell cycle progression of AA8 and UV61 cells (binucleated/mononucleated index) treated with 15µM of 5-azadC for 12h. Cultures were then washed and allowed to recover in fresh media or in media containing DRB (20µM) for 12h. Cultures were washed and allowed to recover in cytochalasin B containing media for 18h, fixed and processed according to the material and methods section. The average and SEM of two independent experiments is shown.

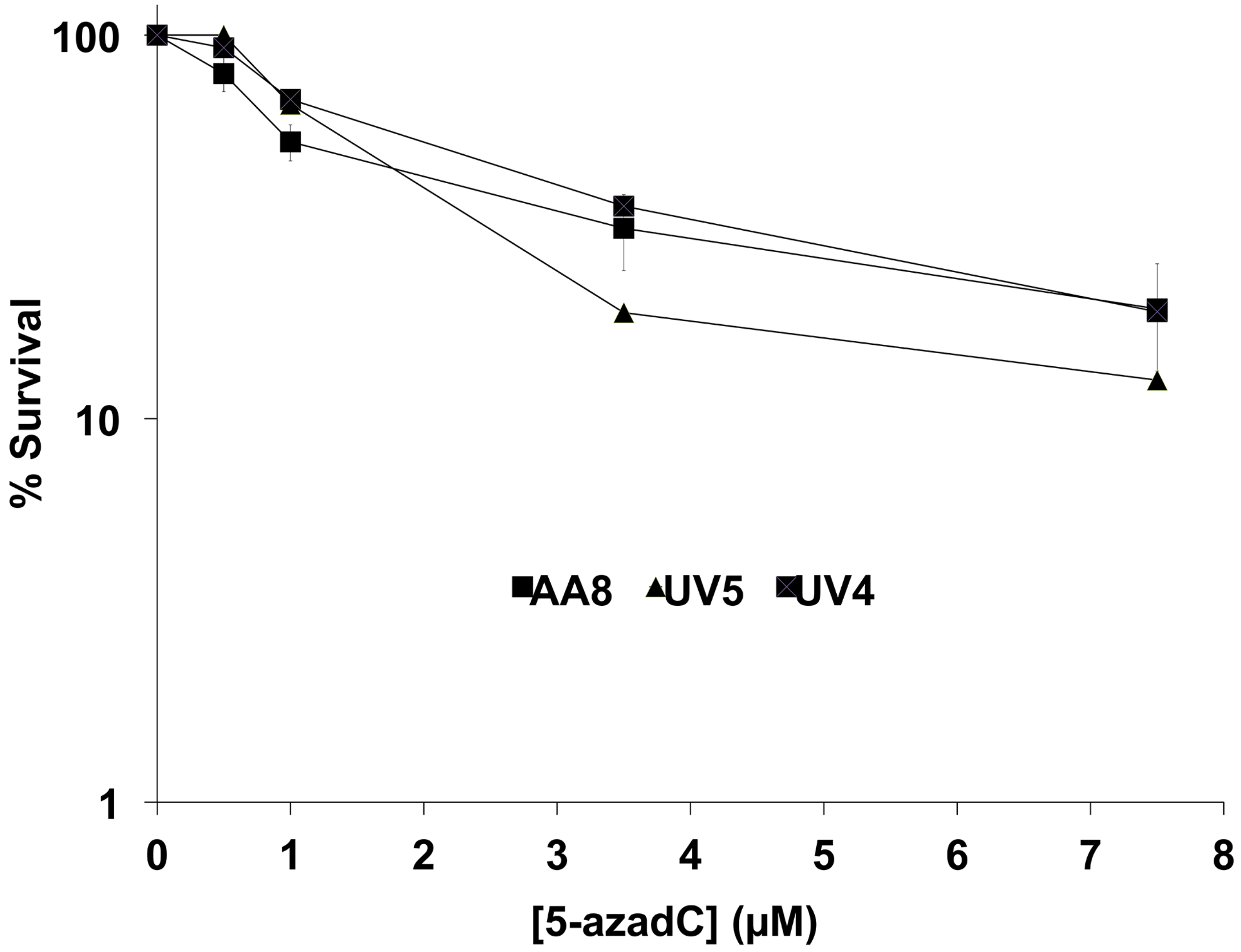

Supplementary Figure 3: ERCC1 and ERCC2 (XPD) are not essential to repair 5-azadC induced lesions. AA8 (wild type), UV4 (ERCC1deficient) and UV5 (XPD deficient) were seeded at low density and treated with increasing doses of 5-azadC for 24h. Cultures were then washed and cells were allowed to form colonies for 7-9 days. Cultures were processed as described in the material and methods section. The average and SEM of two independent experiments is shown.
